# Dirt

**Published:** 1882-08

**Authors:** 


### DIRT.

FILTH, disease, and moral death are associated together in all times and places; and one of the most direct, efficient and speedy means of promoting the physical health of individuals and communities is to bring them under the influence of sound moral and religious principles, through the instrumentality of the Bible, the pulpit, and properly conducted Sundayschools and Sunday reading. True religion not only purifies the sentiments and the heart, but washes a man's clothes, neatly patches every worn-out garment, keeps his house or farm in good repair, and inaugurates system, promptitude, exactness, courtesy, and a spirit of generous forbearance and kindly accommodation in every household. It is not intended to say there is no piety where these things are not found, but they most certainly do abound in proportion as Christianity has her "perfect work," hence the articles which first follow are not out of place in a Journal of Health. No system of hygiene can be complete which does not include that temperance, industry, and personal purity which the sacred writings often and strongly insist upon.

